# Can Neurocritical Care Guidelines Developed in High-Income Countries be Relevant to Low- and Middle-Income Countries?

**DOI:** 10.1007/s12028-025-02292-3

**Published:** 2025-05-29

**Authors:** Venkatakrishna Rajajee

**Affiliations:** Department of Neurosurgery, University of Michigan, Ann Arbor, MI USA

**Keywords:** Guideline, Low and middle, Income countries, Critical care, Cost analysis, Acute brain injuries

## Abstract

Guideline-concordant care of patients with brain injury may improve outcomes. Guidelines developed in high-income countries (HICs) may overlook important considerations in low- and middle-income countries (LMICs), where resources are often constrained. Many LMICs lack resources for guideline development. Professional societies in HICs can achieve greater worldwide impact through a focus on LMIC concerns during guideline development. Guideline panels that address LMIC concerns should include experts from LMICs and frame population, intervention, comparison, and outcome (PICO) questions appropriate to these settings. The greatest challenge to LMIC-focused guidelines is the paucity of high-quality evidence. Recommendations may be weak or conditional because of reliance on indirect data and observational studies. Methods to evaluate cost-effectiveness developed by the World Health Organization may be useful in addressing the value of system-based interventions such as trauma centers. However, marked variability exists within LMICs in the ability of individuals and centers to access and afford treatments. It is challenging, therefore, for guidelines panels to recommend that expensive but potentially beneficial individual treatment options considered appropriate in HICs not be used in LMICs based on cost considerations alone. Rationing of intensive care unit beds and other resources is unfortunately common in resource-constrained regions. LMIC-focused guidelines on accurate prognostication may allow for better-informed counseling of surrogates and goals of care discussions. This may prevent potentially devastating out-of-pocket expenditure when the probability of meaningful recovery is low. A useful approach to LMIC-focused neurocritical care PICOs may be to posit a situation in which the expensive but potentially beneficial intervention is unavailable, and the question is framed around feasible alternatives in the local environment. This will allow for the possibility that the preferred expensive intervention may be available in some LMIC settings but unavailable in others. Examples include alternative management strategies when tools such as invasive intracranial pressure monitoring or continuous electroencephalography are unavailable. Guidelines that consider LMIC-specific concerns are feasible and may improve the care of critically ill patients with neurological illness worldwide.

- *In 2006, a 66-year-old man in a lower middle-income country developed septic shock and multiorgan dysfunction. His intensivist was aware that the 2004 Surviving Sepsis guidelines recommended the use of the drug drotrecogin alfa in this situation *[1]*. The patient’s family consented to its use after they were counseled on the potential mortality benefit. However, all their health care expenses were out-of-pocket, and the drug cost $10,000, over and above all other expenses. This exhausted their savings and required a loan. The patient died after a prolonged intensive care unit (ICU) stay. A subsequent clinical trial did not confirm the benefit of drotrecogin alfa, and the drug was withdrawn *[2, 3]*.*
- *A 25-year-old man with severe traumatic brain injury (sTBI) and a Glasgow Coma Scale (GCS) score of E1-V1T-M4 is admitted to a neurocritical care unit (NCCU) in a lower middle-income country. Although intracranial pressure (ICP) monitoring is recommended by Brain Trauma Foundation (BTF) guidelines *[4, 5]*, this is unavailable at the institution. The patient has cerebral contusions, cerebral edema, and 7 mm of midline shift. The neurosurgeon and intensivist discuss the options: maximize medical management with serial neurological examinations and computed tomography (CT) scans, use ultrasound-based noninvasive ICP assessment, or perform a primary decompressive hemicraniectomy.*
- *A 65-year-old man is rushed to a hospital in a low-income country with sTBI and a GCS score of E1-V1-M2. The only ICU in the region has 15 beds. Only one bed is available. The labor and delivery ward simultaneously requests a bed for a 21-year-old woman with postpartum hemorrhage and shock. Neuroprognostication guidelines suggest that neither age nor GCS score in this ultra-early period reliably predicts outcome *[6]*. However, the patient with sTBI is denied admission because his likelihood of survival with good outcome is perceived as poor compared to the obstetric patient.*

## Introduction

The first two scenarios describe patients cared for by the author, whereas the third describes a hypothetical but unfortunately common scenario in severely resource-constrained regions [7]. Other than being common situations in low- and middle-income countries (LMICs), these case scenarios share another common factor: guideline panels in high-income countries (HICs) do not routinely consider scenarios such as these when they formulate recommendations. Guidelines play a critical role in health care delivery. Compliance with guidelines for the management of sTBI and stroke likely improves long-term outcomes and decreases resource use [8–14]. However, critically ill patients in LMICs may not realize the benefits of guideline-concordant care to the same extent as patients in HICs because guidelines developed in HICs may overlook key considerations in a very different, typically resource-constrained, environment. Although guidelines with an LMIC focus have been developed in fields such as primary care and perioperative management [15, 16], there is a shortage of LMIC-focused critical care guidelines from international organizations. The importance of critical care in LMICs is often underappreciated compared to health care interventions such as preventive care [17–19]. Guidelines may have the greatest impact in LMICs, where highly trained specialists such as neurointensivists are scarce [15, 16]. Because the burden of caring for complex critically ill patients often falls on providers with varied training, a focus on guideline-concordant practice can ensure a minimum standard of care. The objective of this article is to review some of the major considerations and challenges in the development of neurocritical care guidelines applicable to LMICs. Key considerations in guideline development, the role of cost-effectiveness, the importance of prognostic guidelines, and a suggested approach to formulating LMIC-focused guideline questions will be discussed.

## Guideline Panels and Clinical Questions

Several high-quality guidelines have originated from critical care societies in LMICs [20–22]. Guideline panels convened by these societies benefit from direct knowledge of two key evidence-to-recommendation criteria of the Grading of Recommendations Assessment, Development, and Evaluation (GRADE) system, as applicable to the local environment: values and preferences and resource considerations [23, 24]. Critical care societies in several LMIC regions, however, lack the resources to independently develop guidelines. Guideline panels convened by professional societies in HICs are better resourced and are well-positioned to consider LMIC scenarios while formulating recommendations. First, guidelines written by these societies, such as the Surviving Sepsis and BTF guidelines, often frame the standard of care far beyond their countries of origin [5, 25]. Second, the membership of these societies has become international to a greater degree than ever before [26]. It is in the interest of these societies to cater to and grow their membership base, which leads to greater income and worldwide impact. Key considerations for guideline panels in HICs that address LMIC concerns include the following:

- Guideline panels must include qualified individuals from LMICs.
- Population, intervention, comparison, and outcome (PICO) guideline questions should, where relevant, separately address LMIC scenarios. A question directed at fluid bolus administration in sepsis may not require a separate PICO for LMIC settings, but a PICO directed at expensive technology or medications might.
- A less resource-intensive alternative to writing new guidelines is to adapt existing guidelines for LMIC settings because only select guideline questions may need to be changed.

## Evidence-Based Guidelines or Consensus Statements?

The greatest challenge in the development of LMIC-specific guideline recommendations is the paucity of high-quality evidence from LMICs, particularly randomized controlled trials (RCTs). Guideline development systems such as GRADE place a premium on RCTs and high-quality evidence [23, 27]. An alternative approach is to focus on expert opinion and consensus. This approach has important limitations. Recommendations may be skewed by individual practice preferences, whereas other approaches may be equivalent or superior. Replacing a structured systematic review with an unstructured review of evidence may lead to important publications being overlooked. This is especially a concern with publications from LMICs. Studies suggest explicit bias in the editorial and peer-review process in high-impact journals against publications originating from LMICs, when all other factors are equal [28–31]. In the absence of a librarian search and a standardized risk-of-bias assessment tool, expert panels may not objectively evaluate studies from LMICs, which may be preferentially published in lower-impact journals. High-impact guidelines developed using a structured process such as GRADE are feasible in the absence of high-quality direct evidence. The March 2020 guidelines on the management of critically ill adults with coronavirus disease 2019 (COVID-19), for example, provided clinical guidance on a completely new disease despite the presence of only minimal direct evidence [32]. Such guidelines rely on indirect evidence from analogous conditions and settings. A body of evidence originating predominantly from HICs but applied to an LMIC-directed PICO may, in some situations, require a downgrade for indirectness [23, 33]. Greater reliance on surrogate outcomes and observational studies may be unavoidable. Comparative effectiveness research from large registries with prospective data entry can provide useful observational data on the efficacy of interventions such as ICP monitoring in real-world LMIC settings [34]. Recommendations are likely to be weak or conditional, which is appropriate where certainty in the evidence is low. Panels should, however, commit to making a recommendation in one direction or the other because a guideline panel will have greater expertise and knowledge of the existing evidence than most clinicians seeking guidance [23, 35]. Guideline development in critical care using a structured process is therefore feasible and should be the standard, as it has been in HICs.

## Resource Constraints and Cost-Effectiveness

Consideration of cost-effectiveness is critical in LMICs but is often missing from HIC-focused guidelines. Resource use is a GRADE evidence-to-recommendation criterion and may carry greater weight for some LMIC-focused PICO questions. Cost-effectiveness is ideally judged in terms of cost per disability-adjusted life-year. The World Health Organization provides guidance on cost-effectiveness and priority setting in LMICs [36]. On the basis of such guidance, some LMIC trauma centers, for example, have been categorized as “very cost-effective” [37, 38]. NCCUs may similarly prove highly cost-effective in LMICs relative to HICs given the high burden of treatable diseases and the lower overall cost of care. Several challenges exist, however, with the use of cost-effectiveness to weigh the direction and strength of recommendations. There are relatively few cost-effectiveness studies from LMICs in critical care. In the absence of more rigorous cost-effectiveness assessment, crude measures of resource use are sometimes substituted, such as the cost of specific interventions or the impact of an intervention on days of mechanical ventilation or length of stay. Guideline panels should be cautious in making any determination that an intervention appropriate in HICs is not appropriate in LMICs based on intervention cost or resource use alone.

There is much heterogeneity among LMICs and within countries. An important consideration is variability in out-of-pocket expenses based on the availability of third-party payers and government-sponsored health care. Out-of-pocket expenditure as a percentage of current health expenditure is greater in LMICs than HICs, especially in Southeast Asia and Africa [39]. Fortunately, out-of-pocket expenditure has seen a steady decline between 2000 and 2021 in some LMICs [39]. Per-capita income has also risen in many LMICs in the last several decades [40].

The first case scenario described illustrates the difficulty with a one-size-fits-all approach to LMIC-focused recommendations. Following a recommendation for the use of drotrecogin alfa in the 2004 Surviving Sepsis guidelines, the drug was made available in several LMICs despite questions about its role in these settings [41]. An analogous situation currently exists with drugs such as andexanet alfa for the reversal of factor Xa inhibitors in patients with intracranial hemorrhage [42]. An expensive, potentially life-saving medication with lower certainty of benefit may have a lesser impact on personal finances in LMICs when a patient is covered by a third-party payer or the family has considerable resources. In contrast, large out-of-pocket expenses may be financially devastating to a family with limited resources and are associated with poverty in LMICs [43, 44]. Such individual variations make it challenging to decide the direction or strength of LMIC-focused recommendations purely on the basis of intervention cost. In contrast, recommendations that address larger-scale interventions by LMIC health care systems, such as the establishment of trauma centers or NCCUs, are feasible based on robust region-specific analyses of cost-effectiveness [36]. An additional factor is the potential for lower-cost alternatives to expensive interventions. In the second case scenario, for example, the availability of an innovative low-cost ICP monitor [45] may allow for BTF guideline-concordant care and render the other choices unnecessary.

## Prognostication and Rationing

The third case scenario describes rationing of critical care resources, specifically an ICU bed. Although health care providers in HICs were forced to ration critical care resources during the COVID-19 pandemic, this has long been a feature of medical care in LMICs. A discussion of the ethics of rationing is beyond the scope of this article [46–48]. However, salvageability, or the likelihood that a patient will recover with treatment, has long been a de facto basis for triage. The reason to deny an ICU bed in this hypothetical scenario was not the patient’s age alone; it was instead the patient’s perceived prognosis. Accurate prognostication can, in theory, allow for better allocation of highly constrained resources, not just through the more controversial process of rationing but by allowing families to make informed decisions on goals of care. Such decisions may be especially important when prolonged ICU care will result in large out-of-pocket expenses in an LMIC setting, with a low probability of meaningful recovery.

The limited availability of rehabilitative services and post-ICU care in several LMIC settings may further restrict the potential for recovery. Prognostication guidelines may therefore be especially relevant to LMICs. With some exceptions, single prognostic variables, such as age, are not reliable in neuroprognostication [49–52]. Multivariate clinical prediction models, such as CRASH, which provides country-specific probabilities of death and unfavorable 6-month outcome, have therefore been developed in conditions such as sTBI [6, 53, 54]. Guideline panels should address the reliability of these models specifically in LMIC settings. This task is especially challenging in neurocritical care because an inaccurate assessment of poor outcome may lead to withdrawal of life-sustaining therapy in a patient who might otherwise have made a good recovery. The majority of existing models in the field of neurocritical care therefore have not met the threshold for reliability [6, 49–52]. Including local patient/family representatives and LMIC clinicians as part of the guideline panel is essential to provide insight on values and preferences. Representatives from LMICs may also help guide recommendations on the appropriate period of supportive care and observation for neurological improvement when the prognosis is indeterminate. Recent guidelines acknowledge this period may vary in resource-constrained regions compared to better-resourced settings [49]. Although resource constraints often dictate the period of supportive care, this does introduce ethical concerns in some patients with severe brain injury. Beneficence, a low probability of functional recovery with ongoing supportive care, must sometimes be weighed against the patient’s suffering as well as the potentially devastating financial impact on caregivers, who may forego education or employment to care for the patient.

## Approach to LMIC-Specific Guideline Questions in Neurocritical Care

When an expensive intervention has demonstrated benefit in an HIC, it is challenging for a guideline panel to recommend against its use in LMICs, even when uncertainty exists in the magnitude of benefit. An alternative approach may be to posit a situation in which the expensive but potentially beneficial intervention is unavailable in a specific setting and frame the question around potential alternatives. This allows for the possibility that some centers and individuals in LMICs may in fact have access to the expensive but potentially beneficial intervention but also recognizes the reality that many others will not. A useful example is the use of invasive ICP monitoring following sTBI. Guidelines recommend the use of this tool because there is observational evidence of reduction in mortality [5]. However, there is uncertainty in the benefit of this intervention in LMICs. A clinical trial conducted in Bolivia and Ecuador [55], as well as an international observational study that included conditions other than sTBI [56], did not suggest benefit in these settings. The evidence is not uniform, however. Evidence from a national referral center in India with access to more advanced technology suggests the same mortality benefit as observed in HICs [34, 57]. Although the primary PICO question could address whether or not to use invasive ICP monitoring following sTBI, a corollary PICO question could be “In patients with sTBI, when ICP monitoring is unavailable, should intermittent noninvasive ultrasound-based ICP assessment be performed or should monitoring be based on serial clinical neurological assessment and CT imaging?” This would be an extremely useful question for LMIC settings. A further question could be, for example, “In patients with sTBI with midline shift > 5 mm on CT, when invasive monitoring is unavailable, should a primary decompressive hemicraniectomy be performed versus serial clinical neurological assessment and CT imaging?” Currently, the body of evidence with direct comparison of these alternatives is nonexistent. However, the recognition that these are important and valid guideline questions in LMIC settings may prompt further research and eventual clinical trials. A similar question in patients with status epilepticus could be “In patients who require intubation for status epilepticus, when continuous electroencephalography (EEG) is unavailable, should a single EEG be performed to exclude nonconvulsive status epilepticus versus serial intermittent EEG?”.

## Conclusions

Neurocritical care guidelines should address LMIC-specific scenarios and concerns. Additional PICO questions may address alternate approaches when specific interventions are unavailable because of resource constraints.
